# Development and Validation of an Analytical Method for Determination of Al, Ca, Cd, Fe, Mg and P in Calcium-Rich Materials by ICP OES

**DOI:** 10.3390/molecules26206269

**Published:** 2021-10-16

**Authors:** Anna Szymczycha-Madeja, Maja Welna, Monika Zabłocka-Malicka, Pawel Pohl, Włodzimierz Szczepaniak

**Affiliations:** 1 Division of Analytical Chemistry and Chemical Metallurgy, Faculty of Chemistry, Wroclaw University of Science and Technology, Wybrzeze Wyspianskiego 27, 50-370 Wroclaw, Poland; maja.welna@pwr.edu.pl (M.W.); monika.zablocka-malicka@pwr.edu.pl (M.Z.-M.); pawel.pohl@pwr.edu.pl (P.P.); 2 Department of Water and Wastewater Treatment Technology, Faculty of Environmental Engineering, Wroclaw University of Science and Technology, Wybrzeze Wyspianskiego 27, 50-370 Wroclaw, Poland; wlodzimierz.szczepaniak@pwr.edu.pl

**Keywords:** calcium-rich materials, sample preparation, multi-element analysis, validation, ICP OES

## Abstract

Four procedures based on closed-vessel microwave-assisted wet digestion with different oxidative reagents, including HNO_3_ (P1), HNO_3_ + H_2_O_2_ (P2), *aqua regia* (P3) and *Lefort aqua regia* (P4), for preparation of calcium (Ca)-rich materials prior to determination of total concentrations of Al, Ca, Cd, Fe, Mg and P by inductively coupled optical emission spectrometry (ICP OES) were compared. It was found that digestion with *Lefort aqua regia* (P4) provided the best results for all examined elements, i.e., precision of 0.30–4.4%, trueness better than 2%, recoveries of added elements between 99.5–101.9%, and limits of detection within 0.08–1.8 ng g^−1^. Reliability of this procedure was verified by analysis of relevant certified reference materials (CRMs), i.e., Natural Moroccan Phosphate Rock—Phosphorite (BCR-O32). Additionally, selection of appropriate analytical lines for measurements of element concentrations, linear dynamic ranges of calibration curves and matrix effects on the analyte response were extensively investigated. Finally, the selected procedure was successfully applied for routine analysis of other Ca-rich materials, i.e., CRMs such as NIST 1400 (Bone Ash), CTA-AC-1 (Apatite Concentrate Kola Peninsula) and NCS DC70308 (Carbonate Rock), and six natural samples, such as a dolomite, a phosphate rock, an enriched superphosphate fertilizer, pork bones, pork bones after incineration, and after steam gasification.

## 1. Introduction

Calcium (Ca)-rich materials mainly include phosphorite, apatite, dolomite (sedimentary carbonate rock) and bones. Among them, the most important are those that contain phosphorus (P) in addition to Ca. Phosphorites (sedimentary rocks) and apatite rocks (volcanic sediments) are the two main raw materials used for production of P fertilizers [1,2]. Additionally, due to a high P content, the phosphorite is the most important source of this element for the European industry of phosphoric acid. The dolomite is manly used for production of fertilizers, ceramic and refractory materials [3,4]. In the last years, utilization of bones as fertilizers represents a valuable recycling strategy [5,6]. Bones or other by-products obtained from their rendering industry may serve as an additional source of P. Each one can be divided into two main parts, i.e., inorganic and organic. The inorganic part contains mainly calcium phosphate (Ca_3_(PO_4_)_2_), while the organic part is made of gelatin [7].

Due to the fact that Ca-rich materials are used in industry, elemental characterization is the first and essential step in evaluating their suitability for direct applications. In addition to determining the content of the main constituents, namely Ca, Mg, P, Al and Fe, it is extremely important, especially in the case of phosphorites, to determine traces of Cd. This is because Cd occurs naturally in phosphate rocks, therefore, fertilization can increase the risk of Cd contamination and its transferal to the food chain through direct addition of P fertilizers to the soil. Due to the potential hazard to human health, some countries introduced regulations limiting the amount of Cd that can be present in P-based fertilizers. The *European Commission* (EC) proposed the regulation aimed at reducing Cd in fertilizers to a limit of 60 mg Cd/kg P_2_O_5_ (2020 year). However, this limit will be successively reduced to 40 mg Cd/kg P_2_O_5_ after 3 years (2023 year) and then to 20 mg Cd/kg P_2_O_5_ over a 12-year period (2032 year). The *European Parliament* finally adopted the EC’s proposal but with a longer time: 16 years instead of 12 [8].

Sample preparation before analysis is one of the most important and demanding steps of the analytical process, which influences the quality of the results obtained. Only a few measurement techniques allow analyzing samples in their original form, i.e., without any initial pre-treatment. The selection of a digestion method and a sample pre-preparation procedure depends on the composition of the sample matrix, the type and concentration of analytes as well as the technique used for quantification of elements. Total concentrations of selected elements contained in different samples are mostly determined by flame atomic absorption spectrometry (FAAS), inductively coupled plasma optical emission spectrometry (ICP OES) and inductively coupled plasma mass spectrometry (ICP-MS). Unfortunately, these techniques require samples to be in the form of aqueous solutions. For this purpose, due to a complex matrix of Ca-rich materials along with their physical state (solid samples), the most effective way to prepare them for analysis seems to be alkaline fusion. Indeed, procedures referred to alkaline fusion are predominantly described in the professional literature [9,10,11,12,13]. Despite excellent digestive effectiveness, this method is, unfortunately, time-consuming and can cause analytes losses due to volatility of compounds formed at high temperatures of fusion. What is more, it is associated with contamination of the samples due to long-term processing (a two-stage process including fusion with a suitable flux followed by dissolution of resulted samples) and high background levels during measurements as a result of large amounts of fluxes added to the samples. Undoubtedly, all this significantly affects the quality and reliability of the results obtained when using alkaline fusion. Wet digestion of this type of samples is also often carried out. In different wet digestion procedures, dangerous and hazardous mineral acids or their mixtures, typically HF, HClO_4,_ H_2_SO_4_, HNO_3_+HF, HNO_3_+HClO_4_, HF+HClO_4_ [1,13,14,15,16,17], are used. The conventional open-vessel method of acid digestion is time consuming and often leads to systematic errors and failures. Therefore, microwave-assisted wet digestion in a closed-vessel system, facilitated by the use of various oxidizing acids/mixtures (but without those aforementioned aggressive ones), is currently the most commonly recommended and used sample preparation method. It is fast and effective and can reduce the usage of high amounts of reagents, preventing losses of the analytes and secondary contamination of the samples. Such proceeding exactly fits the principles of ‘Green Chemistry’ that recommends the use of safer chemicals to minimize the potential risk of accidents [18,19,20]. Additionally, a very critical source of errors in quantitative analysis of complex materials by spectrometric methods is the effect of the sample matrix on measured analyte signals. Although matrix effects depend on the qualitative and quantitative composition of tested materials, usually they result in lowering the response of analytes. In the case of wet digestion, resulting sample solutions, beside analytes, also contain the matrix of remaining sample compounds (present in their inorganic forms) and the matrix of acids used for digestion. Most often, the inorganic matrix of Ca-rich materials contains significant amounts of alkaline earth elements (Ca and Mg), alkaline elements (Na and K), P and sometimes Fe and Al. Among them, Ca is regarded as the element that produces the most serious chemical interferences in ICP OES [21,22]. Furthermore, the effect of alkaline earth elements (mainly Ca) on the analyte signal was reported to be more meaningful than this observed for alkaline elements [23,24,25,26,27]. Actually, introduction of sample solutions containing large amounts of Ca into the ICP causes significant signal suppressions (up to 40% or more). This effect can be minimized and/or completely eliminated by using experimentally determined correcting factors, but their reliable determination is tedious.

The aim of this work was to develop and validate a new sample preparation procedure of closed-vessel microwave-assisted wet digestion for determination of Al, Ca, Cd, Fe, Mg and P in Ca-rich materials by means of ICP OES. Four various oxidative reagents for sample preparation, i.e., HNO_3_ (P1), HNO_3_ + H_2_O_2_ (P2), *aqua regia* (P3) and *Lefort aqua regia* (P4) were tested. Suitability of the developed procedure, alternative to usually used alkaline fusion with fluxes or open-vessel wet digestion with hazardous acids or their mixtures, was evaluated in terms of selected figures of merit of the ICP OES method, i.e., appropriate analytical lines for measurements of elements concentrations, linear dynamic ranges of calibration curves, limits of detection of elements, precision and trueness of results. Susceptibility of the ICP OES method combined with newly developed closed-vessel microwave-assisted wet digestion to interferences coming from the most significant sample matrix constituents, i.e., major and minor elements (mainly Ca and P, Na, K, Mg, Al, Fe), was also assessed on reliability of determination of studied elements. The effects caused by single interfering elements, as well as their mixture, were studied in detail. The selected procedure was applied to determine all six elements in several Ca-rich materials, including four relevant certified reference materials (CRMs) and six natural samples. It must also be commented that this is the first report presenting the fully validated procedure of preparation of Ca-rich materials by microwave-assisted closed-vessel wet digestion that is suitable for their accurate (precise and true) multi-element analysis by ICP OES.

## 2. Results and Discussion

### 2.1. Reliability of Tested Sample Preparation Procedures

The validity of the tested wet digestion method with solutions of HNO_3_ (P1), HNO_3_ + H_2_O_2_ (P2), *aqua regia* (P3) and *Lefort aqua regia* (P4) applied to prepare the Natural Moroccan Phosphate Rock CRM (BCR-032) before spectrometric measurement was evaluated by choice of appropriate analytical lines and assessing linear dynamic ranges (LDRs) of calibration curves, matrix effects, limits of detection (LODs), precision and trueness of results.

The most prominent analytical lines of the studied elements were selected. The LDRs of calibration curves were assessed for these lines on the basis of a series of simple standard solutions that were used to calibrate the ICP OES instrument. Evaluated optimal concentration ranges guaranteed obtaining reliable analytical results. The linearity of the calibration curves was assessed using determination coefficients (R^2^). Interference effects coming from sample matrix constituents (mainly Ca) on determination of studied elements were examined by evaluating ratios of the intensity of analytical lines measured for their standard solutions containing potential interferents to the intensity of these lines measured for respective standard solutions without any interferents at the same analyte concentration. In these experiments, simple aqueous standard solutions were used, while interfering components reflected composition of real sample solutions analyzed here and selected for optimization study, i.e., the BCR-032 CRM. LODs of studied elements achievable with ICP OES combined with examined sample preparation procedures P1-P4 were calculated according to the 3σ criterion, using the 3σ/a formula, where “a” is the slope of the calibration curve, “σ”—the standard deviation for 10 independent measurements of procedural blank solutions. In the case of evaluation of precision and trueness of determined concentrations of studied elements, suitable statistical tests were applied. Significance of differences between precision of mean concentrations of elements, as expressed by standard deviations (SDs), obtained with compared procedures and precision of certified values of the BCR-032 CRM were tested at the 95% significance level (*p* = 0.05) using the one-tailed Snedecor–Fisher *F*-test with a critical value of this test (*F*_critical_) equal to 19.00 [28]. Precision of these results was additionally expressed as the relative standard deviation (%RSD). Trueness of mean concentrations of elements determined with tested sample preparation procedures was verified through their statistical comparison with assigned certified values of the BCR-032 CRM. When there was no statistically significant differences (*F*_calculated_ < *F*_critical_) between SDs of compared results, the one-tailed Student’s *t*-test was used to compare determined mean concentrations of elements with respective certified values. The 95% significance level (*p* = 0.05) was assumed, for which the *t*_critical_ value was 4.303 [28]. Additionally, the standard addition method was used to evaluate possible matrix effects. For this purpose, prepared sample solutions were spiked with studied elements at two different levels and analyzed by ICP OES to evaluate respective recoveries of these elements.

#### 2.1.1. Analytical Lines and Linear Dynamic Range (LDR)

In the case of spectrometric measurements by ICP OES, selection of appropriate analytical lines and optimal concentration ranges of calibration solutions that guarantee a linear relationship between the signal and the concentration of the analyte in the selected measurement range is very important. The analytical line should have suitable sensitivity and be free from spectral interferences to provide a reasonably high signal-to-noise ratio and, as a result, a suitable LOD value of a given element. Selection of appropriate analytical lines (among those recommended by the apparatus manufacturer) and respective LDRs for Al, Ca, Cd, Fe, Mg and P measurements was made in reference to sensitivity of these lines (a) and determination coefficient (R^2^) of calibration curves for a series of standard solutions of studied elements (see Table 1).

For Cd, out of three tested analytical lines (214.4, 226.5 and 228.8 nm), the 228.8 nm line was excluded due to twice lower sensitivity as compared to these for two other lines. In the case of 214.4 and 226.5 nm lines, the 214.4 nm line was preferred. This line, despite comparable sensitivity to sensitivity of the 226.5 nm line, provided a lower LOD value given in Table 3 (see Section 2.1.3). It was particularly important in the case of determination of traces of Cd in analyzed sample solutions. Considering trace concentrations of Cd, all measurements were made using calibration curves ranging from 0.1 to 2.0 mg kg^−1^ (LDR). In the case of P, out of two tested analytical lines (177.4 nm and 213.6 nm), the 213.6 nm line was favored. Despite high values of R^2^, evidencing linearity of calibration curves for both lines, it was observed that their sensitivities varied and increased with the increasing concentration of P in calibration standard solutions. Accordingly, the difference between slopes of the calibration curves acquired in the range of 0.5–5.0 and 0.5–10 mg kg^−1^ was about 15%. Considering the level of P in prepared sample solutions of the BCR-032 CRM introduced into the ICP spectrometer (including 1000-fold dilution), the recommended working range of calibration curves for P was 0.5–5.0 mg kg^−1^ instead of 0.5–10 mg kg^−^^1^. Consequently, this range was chosen for further studies. Similarly as for P, the Ca content was possible to be determined in 1000-fold diluted sample solutions, hence, a more sensitive analytical line (317.9 nm) was chosen for measurements. The evaluated LDR was, depending on the content of this element in prepared sample (BCR-032 CRM) solutions, within 0.5–5.0 mg kg^−1^ or 0.5–10 mg kg^−1^. For Al and Fe, 396.1 nm (Al) and 238.2 nm (Fe) lines were more advantageous than other tested lines of these elements due to much better sensitivities. The only exception was Mg, for which a less sensitive atomic line (285.2 nm) was the most-liked. This line had a better R^2^ value and provided a lower LOD value than these assessed for the 280.2 nm line. The LDR of calibration curves for these three elements (Al, Fe, Mg) was in the range from 0.5 to 5.0 mg kg^−1^.

#### 2.1.2. Matrix Effects

The matrix of the examined material (mainly Ca) can make considerable problems in determination of other elements, especially those present at a trace level. To check the effect of the real sample matrix on determination of studied elements versus various analytical lines, sets of synthetic solutions, i.e., standard solutions containing 0.05 and 0.1 mg kg^−1^ of Cd, and 1.0 and 2.0 mg kg^−1^ of Al, Fe and Mg in the presence of potential interferents (separately added or combined) were prepared and analyzed. Additionally, to overcome or reduce matrix effects, for elements present in the examined material in large amounts (P, Ca), dilutions of prepared sample solutions before measurement were applied.

In the case of P, independently of its analytical line, 1000-fold dilution of prepared solutions of the BCR-032 CRM samples before measurements allowed to completely eliminate any potential interferents coming from Ca, Na, K, Mg, Al, and Fe on P determination. Additionally, these sample solutions were diluted 100-, 200- and 500-fold. It was observed, that 100- and 200-fold dilutions caused negative errors in determination of P. This effect came from the Ca level present in less diluted sample solutions. Similarly as for 1000-fold dilution, 500-fold dilution resulted in eliminating the interfering effect in Ca on P determinations. Quantitative recoveries obtained for the standard addition method in this case proved no sample matrix effects for 500- and 1000-fold dilutions of original sample solutions. These results confirmed that appropriate dilutions of sample solutions reduce the detrimental effect of matrix components. Simultaneously with P, in 1000-fold diluted sample solutions, the content of Ca was determined, importantly without any negative impact coming from the aforementioned element.

Due to a low Cd content expected to be in all real samples examined in this work (several mg kg^−1^), prepared solutions of the BCR-032 CRM samples were directly introduced (without dilution) into the ICP. For this reason, the effect of six synthetic matrices, reflecting the composition of differently prepared sample solutions of the Natural Moroccan Phosphate Rock CRM (BCR-032), was studied on determination of 0.05 and 0.1 mg kg^−1^ of Cd, including the matrix A: Ca-3500 mg kg^−1^, P-1300 mg kg^−1^; matrix B: Na and K, both at 100 mg kg^−1^; the matrix C: Mg-20 mg kg^−1^; the matrix D: Al-25 mg kg^−1^; the matrix E: Fe-15 mg kg^−1^; the matrix F: the sum of all matrices A–E.

As can be seen from Table 2, obtained values corresponded well with outcomes of the study on selection of appropriate analytical lines. Accordingly, the previously selected 214.4 nm Cd line was also found to be the most resistant to matrix effects than other two lines (226.5 nm and 228.8 nm). It was observed that high contents of Ca and P (matrix A) led to a decrease in the response of Cd up to 34%. The presence of Na and K (matrix B) and Mg (matrix C) slightly lowered the response of Cd, i.e., by 2% and 1%, respectively. The effect of the presence of Al (matrix D) and Fe (matrix E) on determination of Cd was also negligible. The effect (−36%) produced by the combined matrix (matrix F) was close to the sum of effects coming from separate matrices, i.e., A+B+C+D+E (−37%).

Measurements of Al, Fe and Mg concentrations required 10-fold dilution of sample solutions, hence, for these elements, potential interferents were limited to Ca, P, Na and K. Accordingly, for standard solutions containing 1.0 and 2.0 mg kg^−1^ of Al, Fe and Mg (single-element standards were used), the presence of three synthetic matrices with 10-times lower concentrations of interferents, i.e., the matrix G: Ca-350 mg kg^−1^, P-130 mg kg^−1^; the matrix H: Na and K, both at 10 mg kg^−1^; the matrix I: the sum of all matrices (G-H), were tested. As shown in Table 2, in the presence of Na and K (matrix H), independently of the analytical line used, no interfering effects on determination of Al, Fe and Mg were observed. In contrast, the presence of Ca and P (matrix G) led to a decrease in the response for Al, Fe and Mg, and the level of interferences was analytical line dependent. However, the lowest reduction of the response, i.e., by about 6% (Al), 3% (Fe) and 8% (Mg), was observed for initially indicated as the most prominent analytical lines of these elements, i.e., 396.1 nm (Al), 238.2 nm (Fe) and 285.2 nm (Mg). For other lines, interfering effects were up to 3-times higher. Similar to Cd, the effect produced by the combined matrix (matrix I) on the response for Al, Fe and Mg was practically the same as the sum of effects coming from separate matrices (G+H). All these results are very important and should be taken into account to overcome errors in determinations of elements (mainly present at trace levels, such as Cd) in Ca-rich materials by ICP OES.

It is worth mentioning here that sensitivity of determination of analytes may also be affected by acids used for sample preparation by wet decomposition. Except for P and Ca, which could be measured by ICP OES against simple aqueous standard solutions (due to high dilution of prepared sample solutions (1000-fold)), for remaining elements, sample solutions were no (Cd) or 10-fold (Al, Fe, Mg) diluted. Therefore, to eliminate matrix effects in determination of Cd, Al, Fe and Mg (derived from oxidative reagent mixtures used for wet digestion in this work) in undiluted and 10-fold diluted sample solutions, measurements of these elements were made based on matrix-matching standard solutions. Respective standards were prepared using appropriate procedural blanks (P1–P4).

#### 2.1.3. Limits of Detection (LODs)

LODs (in ng g^−1^) of elements for measured lines of studied elements and four tested procedures are listed in Table 3.

It can be concluded that the lowest LOD values for Al, Ca, Cd, Fe, Mg and P were obtained for the following lines: 391.6 nm (Al), 317.9 nm (Ca), 214.4 nm (Cd), 238.2 nm (Fe), 285.2 nm (Mg) and 213.6 nm (P). Considering tested procedures for wet digestion of Ca-rich materials (P1–P4), LODs obtained using *Lefort aqua regia* (P4) were the best. Accordingly, LODs of elements assessed using this procedure were changed from 0.08 ng g^−1^ (P) to 1.8 ng g^−1^ (Al). For the procedure P1 (HNO_3_), LODs for Ca, Cd, Fe and Mg (0.27–0.65 ng g^−1^) were close to these obtained for the procedure P4 (0.25–0.60 ng g^−1^). Unfortunately, for remaining elements, i.e., Al and P, their LODs (0.13–3.3 ng g^−1^) were nearly 2-fold higher than those obtained for the procedure P4 (0.08–1.8 ng g^−1^). For two other procedures, i.e., P2 (HNO_3_ + H_2_O_2_) and P3 (*aqua regia*), LODs of all studied elements, except for P, were up to 2- or 3-times higher than those achieved with the procedure P4. In the case of P, its LOD values increased even by 6-times for the procedure P3 and 12-times for the procedure P2. It should be commented that differences in LODs of elements between tested procedures were mainly caused by differences in signal-to-noise ratios acquired for relevant reagent blanks.

Summarizing, taking into account sensitivities of analytical lines of studied elements, their LODs and susceptibility to matrix effects, the following analytical lines were selected for further investigations: 396.1 nm for Al, 317.9 nm for Ca, 214.4 nm for Cd, 238.2 nm for Fe, 285.2 nm for Mg and 213.6 nm for P. The LDR of the calibration standard solutions for all elements, except for Cd, was 0.5–5.0 mg kg^−1^. In the case Cd, its LDR was shorter, i.e., within 0.1–2.0 mg kg^−1^.

#### 2.1.4. Precision

Total concentrations of 6 elements (mean values with their SDs for n = 3) in the Natural Moroccan Phosphate Rock CRM (BCR-032), as determined by ICP OES in sample solutions prepared using different closed-vessel microwave assisted wet digestion procedures (P1–P4), are presented in Table 4. Additionally, certified values assigned for studied elements in the examined CRM, are included in this table.

SDs of results achieved using tested procedures P1–P4 were compared with those for certified values to assess differences in precision of results. The statistical significance of these differences was verified using the *F*-test [28]. Calculated values of the *F*-test (see *F*_calculated_ values in Table 4) were lower than the critical value of this test (*F*_calculated_ < *F*_critical_). This indicated that SDs of mean concentrations of elements determined in the BCR-032 CRM by ICP OES using tested procedures (P1–P4) did not differ in a significant manner from those given for certified values for this CRM. Hence, it was concluded that precision of these results was at the same level.

Considering certified values, RSDs of mean concentrations of studied elements for the Natural Moroccan Phosphate Rock CRM (BCR-032) were within 0.52–11%. The pooled RSD, calculated for all results and reflecting overall precision, was equal to 3.8%. RSDs and overall precision (given in brackets) of results obtained for the analysis of the BCR-032 CRM by ICP OES combined with tested closed-vessel microwave-assisted wet digestion procedures (P1-P4) were within the following ranges: 0.97–6.4% (3.3%) for HNO_3_ (P1), 0.98–6.4% (3.0%) for HNO_3_ + H_2_O_2_ (P2), 1.83–6.5% (3.5%) for *aqua regia* (P3) and 0.30–4.4% (2.2%) for *Lefort aqua regia* (P4). Indeed, the latter procedure (P4) provided the best precision for all studied elements. Precision attained for wet digestion with HNO_3_ (P1) was quite comparable to this achieved with HNO_3_ + H_2_O_2_ (P2). Finally, precision assessed using *aqua regia* (P3) was slightly poorer than this achieved when other procedures (P1, P2 and P4) were used. Comparing RSDs achieved with the procedure P4 to those assigned for certified values (given by the producer), it was noted that practically the same RSDs for Fe and Mg were obtained. Differences between RSDs were found for other elements, i.e., Al, Cd, P and Ca. Accordingly, RSDs obtained after application of *Lefort aqua regia* in the procedure P4 were 3- (Al), 2.3- (Cd), 1.7- (P) and 1.3-times (Ca) lower in comparison to RSDs given for certified values. Differences in precision were mostly due to the analytical methods used for certification of elements in the Natural Moroccan Phosphate Rock CRM (BCR-032). Al (expressed as Al_2_O_3_) was determined using atomic absorption spectrometry (AAS), a gravimetric method with 8-hydroxyquinoline, a spectrophotometric method, X-ray fluorescence (XRF) and neutron activation analysis (NAA). The content of Ca (expressed as CaO) was obtained after applying a volumetric method with KMnO_4_ and titration with EDTA. For determination of P (expressed as P_2_O_5_) the following methods were used: quinoline phosphomolybdate gravimetry, spectrophotometry and XRF.

#### 2.1.5. Trueness

To test the significance of difference between certified mean concentrations of studied elements given in the certificate of the BCR-032 CRM and mean concentrations of these elements achieved using ICP OES combined with sample preparation procedures P1–P4, the *t*-test (*F*_calculated_ < *F*_critical_) was used. When calculated values of the *t*-test were lower than the critical value, i.e., *t*_calculated_ < *t*_critical_, this meant that there was no statistically significant differences in results (means concentrations) obtained using ICP OES with tested procedures (P1–P4) and certified values. In this way, trueness of such results and lack of any systematic error was confirmed. All calculated values of the *t*-test (*t*_calculated_) are also given in Table 4.

As it can be seen, a good agreement between mean concentrations of elements obtained using the procedure P4 (*Lefort aqua regia*) and certified values was observed. In this case, calculated values of the *t*-test for all studied elements were lower than the critical value of this test (*t*_critical_ = 4.303). For the procedure P1 (HNO_3_), a statistically significant difference between mean concentration and the certified value was only established for Al (*t*_calculated_ = 4.619). In fact, a lower concentration of this element was determined. In the case of other elements (Ca, Cd, Fe, Mg and P), values of *t*_calculated_ were lower than the critical value of this test. The procedure P2 (HNO_3_ + H_2_O_2_) neither provided satisfying results for 2 (Al and Ca) out of 6 determined elements. Unfortunately, for the procedure P3 (*aqua regia*), the content of the majority of elements (Al, Ca, Cd and Fe) was observed to be lower than respective certified values. Differences between concentrations of these elements (measured and certified) were statistically significant (*t*_calculated_ > *t*_critical_). Accordingly, this procedure could lead to analytical errors during determination of 4 out of 6 elements investigated here.

In summary, it was concluded that to accurately (precisely and truly) determine Al, Ca, Cd, Fe, Mg and P in Ca-rich materials by ICP OES, samples of these materials should be wet digested in a closed-vessel microwave-assisted system using *Lefort aqua regia* (P4) before their spectrometric measurements. The use of HNO_3_ (P1) or a mixture of HNO_3_ + H_2_O_2_ (P2) for wet digestion of mentioned samples also seems to be justified, however, only in the case of some selected elements, i.e., Ca, Cd, Fe, Mg and P (procedure P1) and Cd, Fe, Mg and P (procedure P2). The procedure with *aqua regia* (P3) was useless because it only enables to determine concentrations of Mg and P.

#### 2.1.6. Recovery Test

Additionally, in order to assess trueness of results as well as to verify the absence of matrix effects, the standard addition method was used. Recoveries of added elements were calculated by analysis of spiked and unspiked sample solutions of the BCR-032 CRM prepared using all tested procedures (P1–P4). Digested samples of the BCR-032 CRM were spiked with standard solutions of studied elements at two concentrations levels (1.0 and 2.0 mg kg^−1^ for Al, Ca, Fe, Mg, P, and 0.05 and 0.10 mg kg^−1^ for Cd in final sample solutions) and subjected to analysis by ICP OES. Because concentrations of Ca and P in analysed material were high (in %), prepared sample solutions were diluted 1000-fold before standards additions. In the case of Al, Fe and Mg, standards of these elements were added to sample solutions that were 10-fold diluted. For Cd, due to its low content, the standard was added to undiluted sample solutions. Calculated recoveries are collected in Table 5.

As can be seen from Table 5, results of the spike-and-recovery test are coincident with the results of the statistical comparison of mean concentrations of elements. It was established that independently of the spiked level, recoveries of all elements were in the range of 85.5–102.5% (P1), 85.2–100.6% (P2), 79.9–100.0% (P3) and 99.5–101.9% (P4). Results achieved for closed-vessel microwave-assisted wet digestion with *Lefort aqua regia* (P4) led to quantitative recoveries of all studied elements. Recoveries obtained for procedures P1 (HNO_3_) and P2 (a mixture of HNO_3_ + H_2_O_2_) indicated that they were not suitable for preparation of samples of Ca-rich materials prior to determination of Al (P1) or Al and Ca (P2) by ICP OES. Results obtained for the procedure P3 (*aqua regia*), similarly as for the statistical comparison, provided quantitative recoveries only for Mg and P.

To sum up, considering important analytical figures of merit investigated here, closed-vessel microwave-assisted wet digestion with *Lefort aqua regia* (P4) was established to be the most advantageous sample preparation procedure prior to determination of Al, Ca, Cd, Fe, Mg and P by ICP OES in the Natural Moroccan Phosphate Rock CRM (BCR-032).

### 2.2. Analytical Application

Finally, to demonstrate the analytical application of the proposed procedure P4 (closed-vessel microwave-assisted wet digestion with *Lefort aqua regia*), it was applied for preparation of different Ca-rich materials, including three CRMs (Bone Ash (NIST 1400), Apatite Concentrate Kola Peninsula (CTA-AC-1) and Carbonate Rock (NCS DC70308)), and six natural samples, such as a dolomite, a phosphate rock, an enriched superphosphate fertilizer, pork bones, pork bones after incineration and pork bones after steam gasification, prior to their multi-element analysis by ICP OES. Determined mean concentrations of Al, Ca, Cd, Fe, Mg and P (*n* = 3) along with respective standard deviations are given in Table 6. Additionally, assigned certified or informative values in CRMs, and values declared for natural products by manufacturers (if available) are included in this table.

Precision (%RSD) of measurements was very good (below 5%) and ranged between: 0.26% (Ca)–0.88% (Mg) in the Bone Ash (NIST 1400 CRM), 0.56% (P)–1.3% (Cd) in the Apatite Concentrate Kola Peninsula (CTA-AC-1 CRM), 0.96% (Fe)–2.5% (P) in the Carbonate Rock (NCS DC70308 CRM), 0.31% (Cd)–2.9% (P) in the dolomite, 0.58% (Ca)–2.6% (Al) in the phosphate rock, 0.58% (P)–3.1% (Mg) in the enriched superphosphate fertilizer, 1.6% (Mg)–4.8% (Cd) in pork bones, 0.58% (Ca, P)–2.8% (Al) in pork bones after incineration, 0.56% (Ca)–4.6% (Cd) in pork bones after steam gasification. Only in three cases, i.e., for Cd (the Bone Ash CRM and pork bones after incineration) and for Al (pork bones), RSDs were slightly higher, i.e., 5.7%, 6.0% and 9.1%, respectively. Considering means concentrations of elements determined in analysed Ca-rich materials (see Table 6), it was established that in most cases obtained results corresponded well to values given in certificates of CRMs or those declared by manufactures of analysed natural products. To show it, the agreement (in %) expressed as ratios between concentrations of elements determined in samples and their certified values (case of CRMs) or declared values by manufacturers (case of natural products), was calculated. As a result, closeness with certified values for Al (97.2%), Ca (99.5%), Fe (98.6%), Mg (99.9%) and P (103%) in the Bone Ash CRM (NIST 1400), Al (100%), Ca (102%), Fe (98.2%) and Mg (102%) in the Apatite Concentrate Kola Peninsula CRM (CTA-AC-1), Al (101%), Ca (100%), Cd (103%), Fe (99.4%), Mg (101%) and P (103%) in the Carbonate Rock CRM (NCS DC70308) were obtained. A significant difference (~17%) was noticed for Cd in the Bone Ash CRM (NIST 1400) due to a non-certified concentration and a low content of this element in the above-mentioned CRM. For elements determined in the Apatite Concentrate Kola Peninsula CRM (CTA-AC-1) such as Cd and P, no certified or informative values were either given. In the case of the phosphate rock, although it was a natural product (not CRM), determined concentrations of studied elements were compared with certified values assigned for the BCR-032 CRM (Natural Moroccan Phosphate Rock). It could be made due to a similar matrix of these two materials. Accordingly, except for Cd, a very good agreement was obtained for Fe (98.8%), Mg (102%), Al (91.1%), Ca (93.8%) and P (95.8%), i.e., for all remaining elements determined in analysed Ca-rich material. For Cd, the determined content of this element was by ~26% lower. Any differences could arise from the origin of these samples, its sampling and particle size taken for experiments. Nevertheless, it was concluded that obtained results confirmed the similarity between matrices of both materials, i.e., the BCR-032 CRM and the natural phosphate rock, hence, the comparison of results made for the phosphate rock was highly justified. For the dolomite, the agreement between values declared by the manufacturer (minimum and maximum range) and those determined were 106 and 98.7% (with the average of 102%) for Ca, and 111 and 91.0% (with the average of 100%) for Mg. For other elements, i.e., Al, Cd, Fe and P, no data were available. In the case of the enriched superphosphate fertilizer, its manufacturer gave the information only about P and Ca contents. Nonetheless, the results for P agreed well (~100%) with the declared value. Unfortunately, for Ca, a 3-fold higher value was attained. This difference could probably be due to the fact that the manufacturer usually provides a minimum indicative content of particular ingredients. Analysing the results achieved for bone samples, i.e., pork bones, pork bones after incineration and pork bones after steam gasification, it was noticed that concentrations of elements determined in pork bones were lower than those in bones after incineration and pork bones after steam gasification. Such tendency was found for Al (3–10-fold), Fe (3-fold) and Ca, Mg, P (6-fold). The only exception was Cd, which content in untreated bones were higher 2–3-fold) than this after incineration and steam gasification.

## 3. Materials and Methods

### 3.1. Instrumentation

An Agilent bench-top optical emission spectrometer (model 720) with an axially viewed Ar-ICP was used for measuring concentrations of Al, Ca, Cd, Fe, K, Mg, Na and P. The instrument was equipped with a high-resolution echelle-type polychromator and a VistaChip II CCD detector. The plasma was sustained in a standard, one-piece quartz torch with an injector tube of 2.4 mm ID. A single-pass glass cyclonic spray chamber (Agilent) and an OneNeb^®^ pneumatic concentric nebulizer (Agilent) were used for introducing solutions of samples and standards by pneumatic nebulization. Operating conditions recommended by the manufacturer were applied, i.e., the RF power: 1200 W; Ar flow rates: 15.0 L·min^−1^ (plasma), 1.5 L·min^−1^ (auxiliary) and 0.75 L·min^−1^ (carrier/nebulizer); the stabilization delay: 15 s; the sample uptake delay: 30 s; the rinse time: 10 s; the replicate read time: 1 s; the number of replicates: 3. The following analytical lines were selected for measurements: 309.1 and 396.1 nm for Al; 315.8 and 317.9 nm for Ca; 214.4, 226.5 and 228.8 nm for Cd; 238.2 and 259.9 nm for Fe; 766.4 nm for K; 280.2 and 285.2 nm for Mg; 589.5 nm for Na; 177.4 and 213.6 nm for P. A fitted background mode with 7 points per a line profile was applied for background correction.

The *Multiwave PRO* microwave reaction system (Anton Paar GmbH, Austria) was used for closed-vessel microwave-assisted wet digestion of samples of CRMs in different reagent mixtures. It was equipped with a 24HVT50 rotor with 50 mL PTFE-TFM pressure-activated-venting vessels.

### 3.2. Reagents and Solutions

EMSURE® ACS grade reagents, i.e., concentrated HNO_3_ (65%, *m*/*v*), HCl (37%, *m*/*v*) and H_2_O_2_ (30%, *m*/*v*) solutions, were purchased from Merck (Merck KGaA, Darmstadt, Germany). *Aqua regia* and *Lefort aqua regia* solutions were freshly prepared by mixing concentrated HCl and HNO_3_ solutions at 3:1 and 1:3 volumetric ratios (*v*:*v*), respectively. De-ionized water (18.3 MΩ·cm) from an EASYpure^TM^ water purification system (Barnstead/Thermolyne Corporation, Dubuque, IA, USA) was used throughout. Commercially available ICP standards, i.e., a Merck Certipur^®^ multi-element stock solution XVI (1000 μg·mL^−1^), single-element stock solutions (1000 μg mL^−1^) of Al, Ca, Cd, Fe, K, Mg, Na, P (all from Merck) and a single-element stock solution (10,000 μg mL^−1^) of Ca (Merck), were used for preparing simple and matrix-matching standard solutions for calibration of the ICP OES instrument.

### 3.3. Samples and Their Preparation

Four relevant Ca-rich CRMs, i.e., BCR-032 (Natural Moroccan Phosphate Rock—phosphorite), NIST 1400 (Bone Ash), CTA-AC-1 (Apatite Concentrate Kola Peninsula) and NCS DC70308 (Carbonate Rock), in addition to six natural samples, i.e., a dolomite, a phosphate rock, an enriched superphosphate fertilizer, pork bones, pork bones after incineration, and pork bones after steam gasification, were used. The BCR-032 CRM was selected for optimization studies, i.e., to develop and validate an appropriate sample preparation procedure.

For closed-vessel microwave-assisted wet digestion of Ca-rich samples, solutions of HNO_3_ (P1), HNO_3_ + H_2_O_2_ (P2), *aqua regia* (P3) and *Lefort aqua regia* (P4) were used. At the optimization study, samples of the BCR-032 CRM (0.25 g) were weighed into PTFE vessels and poured with concentrated reagents, i.e., 6.0 mL of HNO_3_ (P1), 5.0 mL of HNO_3_ and 1.0 mL of H_2_O_2_ (P2), 6.0 mL of *aqua regia* (HCl+HNO_3_, 3:1) (P3) and 6.0 mL of *Lefort aqua regia* (HCl+HNO_3_, 1:3) (P4). Vessels were closed, shielded, inserted into the rotor and subjected to the microwave-assisted heating program with a maximum temperature of 190 °C for 60 min (step 1: 90 °C, 5 min, ramp; step 2: 90 °C, 15 min, hold; step 3: 130 °C, 5 min, ramp; step 4: 130 °C, 15 min, hold; step 5: 190 °C, 5 min, ramp; step 6: 190 °C, 15 min, hold). After cooling and opening the vessels, resulting sample digests were quantitatively transferred into 30-mL PP screw-capped containers (Equimed, Poland) and made up with de-ionized water to 25.0 g. Then, resulting sample solutions were filtered through 0.45 μm membrane syringe filers. Filtrates were collected into 30-mL PP screw-capped containers and refrigerated at 4 °C before their further analysis (ICP OES measurements against solutions of matrix-matching or simple standards). After each mineralization cycle, PTFE digestion vessels were cleaned twice with a HNO_3_:H_2_O (1:1) mixture in order to avoid any contamination due to so-called “memory effect”. For cleaning, the same as for samples mineralization program was used.

All samples were prepared and analysed in triplicate (*n* = 3). Procedural blanks were simultaneously prepared for each sample preparation procedure and considered in final results. Blanks were also used to prepare solutions of matrix-matching standards to compensate any effects coming from remnants of digestion reagents present in final sample solutions obtained using studied preparation procedures P1–P4. All solutions, i.e., standards for calibration and digested samples with their appropriate dilutions required before measurements, were prepared by weighting to avoid differences in solution density.

Concentrations of Ca and P were measured in diluted sample solutions (1000-fold) against simple aqueous standard solutions. Concentrations of Al, Fe and Mg were also determined in diluted sample solutions (10-, 20-, 100- or 200-fold) using matrix-matching (10- and 20-fold) or simple standard solutions (100- and 200-fold). Only concentrations of Cd were measured in undiluted sample solutions versus matrix-matching standard solutions. Five-point or seven-point calibration curves were used for measurements.

## 4. Conclusions

In this work, we evaluated usefulness of the wet digestion procedure that is suitable at the step of sample preparation of Ca-rich materials, i.e., four kinds of CRMs [BCR-032 (Natural Moroccan Phosphate Rock—Phosphorite), NIST 1400 (Bone Ash), CTA-AC-1 (Apatite Concentrate Kola Peninsula) and NCS DC70308 (carbonate rock)] and six natural samples (a dolomite, a phosphate rock, an enriched superphosphate fertilizer, pork bones, pork bones after incineration and pork bones after steam gasification) before determination of total concentrations of Al, Ca, Cd, Fe, Mg and P by ICP OES in all of them. The developed sample preparation procedure of Ca-rich materials, based on closed-vessel microwave-assisted wet digestion in *Lefort aqua regia* (P4), was safe, fast and gave reproducible and reliable results of determination of total concentrations of all six elements. Effects from sample matrix on determination of trace elements were eliminated by using experimentally determined factors. The selected procedure is characterized by very good figures of merit, i.e., precision of results (0.30–4.4%) trueness of results (<2%), recovery of elements (99.5–101.9%) and LODs of elements (0.08–1.8 ng g^−1^). It considerably improves the sample preparation step of Ca-rich material prior to their analysis by ICP OES reducing the reagents consumption and the time of the analysis. Moreover, a much simpler sample handling makes it a vital alternative to alkaline fusion or open-vessel wet digestion with different hazardous acids/mixtures procedures adequate for routine analyses. The use of HNO_3_ (P1) or mixture of HNO_3_ + H_2_O_2_ (P2) for wet digestion of Ca-rich materials, although also useful and justified for most of elements, cannot be used for the determination of Al (P1) and Al and Ca (P2). Unfortunately, digestion with *aqua regia* (P3) was found to be the least efficient and not applicable in practice for multi-element determination in this kind of materials.

## Figures and Tables

**Table 1:** Comparison of the analytical performance of different analytical lines of Al, Ca, Cd, Fe, Mg and P achieved by ICP OES.

Element	Analytical Lines, nm	LDR ^a^	A ^b^	R^2 c^
Al	309.2	0.5–10	8.26 × 10^3^	0.9999
		0.5–5.0	8.50 × 10^3^	0.9998
	396.1	0.5–10	4.18 × 10^4^	0.9999
		0.5–5.0	4.26 × 10^4^	0.9999
Ca	315.8	0.5–10	1.18 × 10^4^	0.9999
		0.5–5.0	1.18 × 10^4^	0.9999
	317.9	0.5–10	3.86 × 10^4^	0.9999
		0.5–5.0	3.86 × 10^4^	0.9999
Cd	214.4	0.1–10	3.94 × 10^4^	0.9991
		0.1–5.0	3.98 × 10^4^	0.9997
		0.1–2.0	4.00 × 10^4^	0.9999
	226.5	0.1–10	3.72 × 10^4^	0.9990
		0.1–5.0	3.73 × 10^4^	0.9998
		0.1–2.0	3.83 × 10^4^	0.9999
	228.8	0.1–10	1.65 × 10^4^	0.9996
		0.1–5.0	1.65 × 10^4^	0.9997
		0.1–2.0	1.72 × 10^4^	0.9999
Fe	238.2	0.5–10	2.28 × 10^4^	0.9999
		0.5–5.0	3.38 × 10^4^	0.9999
	259.9	0.5–10	2.53 × 10^4^	0.9998
		0.5–5.0	2.60 × 10^4^	0.9999
Mg	280.2	0.5–10	1.69 × 10^5^	0.9992
		0.5–5.0	1.78 × 10^5^	0.9996
	285.2	0.5–10	4.35 × 10^4^	0.9998
		0.5–5.0	4.35 × 10^4^	0.9999
P	177.4	0.5–10	8.75 × 10^1^	0.9996
		0.5–5.0	7.39 × 10^1^	0.9982
		5.0–10	1.11 × 10^2^	0.9980
		10–100	1.43 × 10^2^	0.9985
		50–200	1.80 × 10^2^	0.9912
	213.6	0.5–10	1.34 × 10^3^	0.9999
		0.5–5.0	1.16 × 10^3^	0.9999
		5.0–10	1.25 × 10^3^	0.9999
		10–100	1.39 × 10^3^	0.9999
		50–200	1.50 × 10^3^	0.9999

^a^ Linear dynamic range. It refers to the concentration range (in mg kg^−1^) for which an element can be accurately determined. ^b^ Slope of the calibration curve (in (a.u.)/(mg kg^−^^1^)). ^c^ Determination coefficient.

**Table 2:** Matrix effects on determination of Cd, Al, Fe and Mg by ICP OES in presence of potential interferents (separately added and combined).

Analyte	DF ^a^	Matrix	Interferent	Concentrations, mg kg^−1^	Martix Effect, %		
Cd	none				**214.4 nm**	**226.5 nm**	**228.8 nm**
		A	Ca+P	3500 + 1300	−34	−35	−35
		B	Na+K	both at 100	−2	−5	−4
		C	Mg	20	−1	−3	−2
		D	Al	25	none	none	none
		E	Fe	15	none	none	none
		F	Ca+P+Na+K+Mg+Al+Fe	as above	−36	−43	−41
Al	×10				**309.2 nm**	**396.1 nm**	
		G	Ca+P	350 + 130	−19	−6	
		H	Na+K	both at 10	none	none	
		I	Ca+P+Na+K	as above	−19	−6	
Fe	×10				**238.2 nm**	**259.9 nm**	
		G	Ca+P	350 + 130	−3	−4	
		H	Na+K	both at 10	none	none	
		I	Ca+P+Na+K	as above	−3	−4	
Mg	×10				**280.2 nm**	**285.2 nm**	
		G	Ca+P	350 + 130	−15	−8	
		H	Na+K	both at 10	none	none	
		I	Ca+P+Na+K	as above	−15	−8	

^a^ Dilution factor.

**Table 3:** Limits of detection of elements achieved with ICP OES combined with sample preparation by closed-vessel microwave-assisted wet digestion in solutions of HNO_3_ (P1), HNO_3_ + H_2_O_2_ (P2), *aqua regia* (P3) and *Lefort aqua regia* (P4).

Procedure	LOD, ng g^−1^												
	Analytical Lines, nm												
	Al		Ca		Cd			Fe		Mg		P	
	309.3	396.1	315.8	317.9	214.4	226.5	228.8	238.2	259.9	280.2	285.2	177.4	213.6
**P1**	4.9	3.3	0.89	0.27	0.35	0.53	0.44	0.32	0.51	0.82	0.65	2.0	0.13
**P2**	11	3.6	1.6	0.49	0.46	0.67	0.55	0.93	1.2	1.7	1.3	15	1.0
**P3**	6.8	4.6	1.32	0.40	0.38	0.73	0.48	0.51	0.99	1.5	1.2	4.9	0.52
**P4**	3.8	1.8	0.81	0.25	0.33	0.40	0.43	0.29	0.32	0.74	0.60	1.2	0.08

**Table 4:** Total concentrations of elements in samples of the Natural Moroccan Phosphate Rock—Phosphorite certified reference material (BCR-032) prepared using closed-vessel microwave-assisted wet digestion in solutions of HNO_3_ (P1), HNO_3_ + H_2_O_2_ (P2), *aqua regia* (P3) and *Lefort aqua regia* (P4) before spectrometric measurements of resulting sample solutions by ICP OES.

Element	Unit	Certified Value	Determined Value			
			P1	P2	P3	P4
Al expressed as Al_2_O_3_	g kg^−1^	5.5 ± 0.6	4.7 ± 0.3	4.7 ± 0.3	4.6 ± 0.3	5.2 ± 0.2
Ca expressed as CaO	g kg^−1^	518 ± 4	500 ± 8	490 ± 9	485 ± 10	511 ± 3
Cd	mg kg^−1^	20.8 ± 0.7	20.6 ± 0.2	20.4 ± 0.2	19.2 ± 0.6	20.2 ± 0.3
Fe expressed as Fe_2_O_3_	g kg^−1^	2.3 ± 0.1	2.2 ± 0.1	2.1 ± 0.1	2.0 ± 0.1	2.3 ± 0.1
Mg expressed as MgO	g kg^−1^	4.0 ± 0.1	4.2 ± 0.2	4.1 ± 0.1	4.1 ± 0.1	4.1 ± 0.1
P expressed as P_2_O_5_	g kg^−1^	329.8 ± 1.7	331.4 ± 5.1	331.1 ± 5.0	322.8 ± 5.9	330.6 ± 1.0
			***F*_calculated_**			
			**P1**	**P2**	**P3**	**P4**
Al expressed as Al_2_O_3_			4.00	4.00	4.00	9.00
Ca expressed as CaO			4.00	5.06	6.25	1.78
Cd			12.25	12.25	1.36	5.44
Fe expressed as Fe_2_O_3_			1.00	1.00	1.00	1.00
Mg expressed as MgO			4.00	1.00	1.00	1.00
P expressed as P_2_O_5_			9.00	8.65	12.04	2.89
			***t*_calculated_**			
			**P1**	**P2**	**P3**	**P4**
Al expressed as Al_2_O_3_			*4.619*	*4.619*	*5.196*	2.598
Ca expressed as CaO			3.897	*5.389*	*5.716*	4.041
Cd			1.732	3.464	*4.619*	3.464
Fe expressed as Fe_2_O_3_			1.732	3.464	*5.196*	0.000
Mg expressed as MgO			1.732	1.732	1.732	1.732
P expressed as P_2_O_5_			0.543	0.450	2.055	1.386

The critical value of the *F*-test (*F*_critical_): 19.00 (*p* = 0.05); The critical value of the *t*-test (*t*_critical_): 4.303 (*p* = 0.05). The *t*_calculated_ values higher than the critical value of the *t*-test (*t*_calculated_ > *t*_critical_) are italicized and underlined.

**Table 5:** Recoveries of Al, Ca, Cd, Fe, Mg and P in samples of Natural Moroccan Phosphate Rock—Phosphorite (BCR-032) prepared with microwave-assisted closed-vessel digestion with solutions of HNO_3_ (P1), HNO_3_ + H_2_O_2_ (P2), *aqua regia* (P3) and *Lefort aqua regia* (P4) prior to ICP OES detection.

Analyte	DF ^a^	Added, mg kg^−1^	Recovery, %			
			P1	P2	P3	P4
Al	×10 ×10	1.0	85.5 ± 4.2	85.2 ± 5.3	82.3 ± 7.7	99.9 ± 0.9
		2.0	89.1 ± 3.6	88.4 ± 3.8	86.7 ± 3.9	100.0 ± 0.7
Ca	×1000 ×1000	2.0	102.8 ± 3.2	87.8 ± 12	81.3 ± 9.8	101.9 ± 0.5
		4.0	99.3 ± 2.0	94.8 ± 9.9	83.4 ± 7.3	99.5 ± 0.3
Cd	nonenone	0.05	101.4 ± 1.1	96.0 ± 6.3	79.9 ± 10	99.6 ± 1.3
		0.10	99.6 ± 0.3	96.2 ± 9.0	84.2 ± 9.0	100.2 ± 0.3
Fe	×10 ×10	1.0	101.7 ± 1.1	98.7 ± 2.6	93.5 ± 3.1	99.7 ± 0.9
		2.0	100.3 ± 1.0	98.8 ± 1.8	94.6 ± 2.7	100.1 ± 0.7
Mg	×10 ×10	1.0	102.5 ± 0.5	97.6 ± 1.1	99.9 ± 1.6	99.8 ± 0.4
		2.0	98.3 ± 0.4	100.6 ± 1.0	100.0 ± 1.2	100.9 ± 0.2
P	×1000	1.0	100.2 ± 0.2	100.6 ± 0.3	99.6 ± 1.0	100.0 ± 0.5
	×1000	2.0	99.6 ± 1.1	99.5 ± 0.7	99.0 ± 0.5	100.2 ± 0.6

^a^ Dilution factor.

**Table 6:** Concentrations ^a^ of Al, Ca, Cd, Fe, Mg and P in examined Ca-rich materials (CRMs and natural products) obtained by ICP OES after closed-vessel microwave-assisted wet digestion with *Lefort aqua regia* (P4).

Samples	Concentration					
	Al, g kg^−1^	Ca, g kg^−1^	Cd, mg kg^−1^	Fe, g kg^−1^	Mg, g kg^−1^	P, g kg^−1^
**NIST 1400 (Bone Ash)**	0.515 ± 0.004	380 ± 1	0.035 ± 0.002	0.651 ± 0.001	6.83 ± 0.06	177 ± 1
Value from certificate	0.530 ^iv^	382 ± 1	0.03 ^iv^	0.660 ± 0.027	6.84 ± 0.13	179 ± 2
**CTA-AC-1 (Apatite Concentrate Kola Peninsula)**	4.12 ± 0.03	332 ± 2	0.313 ± 0.004	4.91 ± 0.06	0.442 ± 0.005	159 ± 1
Value from certificate	4.10 ^iv^	327 ± 31	no data	5.00 ^iv^	0.435 ^iv^	no data
**NCS DC70308 (Carbonate Rock)**	0.964 ± 0.018	272 ± 5	0.402 ± 0.010	3.11 ± 0.03	91.2 ± 1.2	0.040 ± 0.001
Value from certificate	0.953 ± 0.105	272 ± 1	0.390 ± 0.12	3.13 ± 0.06	90.2 ± 0.4	0.039 ± 0.008
**Dolomite**	0.650 ± 0.014	226 ± 3	9.67 ± 0.03	7.23 ± 0.06	121 ± 1	0.171 ± 0.005
Value from manufacturer	no data	214–229	no data	no data	109–133	no data
**Phosphate rock ^b^**	2.65 ± 0.07	347 ± 2	15.4 ± 0.4	1.59 ± 0.03	2.47 ± 0.06	138 ± 1
Value from BCR-032 ^c^	2.91 ± 0.32	370 ± 3	20.8 ± 0.7	1.61 ± 0.07	2.41 ± 0.06	144 ± 1
**Enriched superphosphate fertilizer**	4.22 ± 0.07	214 ± 4	8.33 ± 0.22	3.98 ± 0.06	5.20 ± 0.16	172 ± 1
Value from manufacturer	no data	71.5	no data	no data	no data	175
**Pork bones**	0.011 ± 0.001	51.0 ± 1.2	0.413 ± 0.020	0.055 ± 0.002	1.25 ± 0.02	27.6 ± 1.1
**Pork bones after incineration**	0.036 ± 0.001	344 ± 2	0.218 ± 0.013	0.155 ± 0.002	7.56 ± 0.05	171 ± 1
**Pork bones after steam gasification**	0.111 ± 0.002	308 ± 2	0.154 ± 0.007	0.186 ± 0.005	8.08 ± 0.07	177 ± 4

^iv^ Information value (non-certified concentration). ^a^ Real sample analysis (matrix effects included). ^b^ Real sample of the Moroccan phosphate rock. ^c^ Certified Reference Material: Natural Moroccan Phosphate Rock—Phosphorite.

## Data Availability

Data is contained within the article.
